# High-order replica bands in monolayer FeSe/SrTiO_3_ revealed by polarization-dependent photoemission spectroscopy

**DOI:** 10.1038/s41467-021-24783-5

**Published:** 2021-07-28

**Authors:** Chong Liu, Ryan P. Day, Fengmiao Li, Ryan L. Roemer, Sergey Zhdanovich, Sergey Gorovikov, Tor M. Pedersen, Juan Jiang, Sangjae Lee, Michael Schneider, Doug Wong, Pinder Dosanjh, Frederick J. Walker, Charles H. Ahn, Giorgio Levy, Andrea Damascelli, George A. Sawatzky, Ke Zou

**Affiliations:** 1 Quantum Matter Institute, University of British Columbia, Vancouver, BC Canada; 2 Department of Physics and Astronomy, University of British Columbia, Vancouver, BC Canada; 3 Canadian Light Source, Saskatoon, SK Canada; 4 Department of Applied Physics and Center for Research on Interface Structures and Phenomena, Yale University, New Haven, CT USA; 5 Department of Physics, Yale University, New Haven, CT USA

**Keywords:** Electronic properties and materials, Superconducting properties and materials, Surfaces, interfaces and thin films

## Abstract

The mechanism of the enhanced superconductivity in monolayer FeSe/SrTiO_3_ has been enthusiastically studied and debated over the past decade. One specific observation has been taken to be of central importance: the replica bands in the photoemission spectrum. Although suggestive of electron-phonon interaction in the material, the essence of these spectroscopic features remains highly controversial. In this work, we conduct angle-resolved photoemission spectroscopy measurements on monolayer FeSe/SrTiO_3_ using linearly polarized photons. This configuration enables unambiguous characterization of the valence electronic structure with a suppression of the spectral background. We consistently observe high-order replica bands derived from various Fe 3*d* bands, similar to those observed on bare SrTiO_3_. The intensity of the replica bands is unexpectedly high and different between *d*_xy_ and *d*_yz_ bands. Our results provide new insights on the electronic structure of this high-temperature superconductor and the physical origin of the photoemission replica bands.

## Abstract (web summary)

The origin of the photoemission replica bands in monolayer FeSe/SrTiO_3_ remains controversial. Here, the authors perform angle-resolved photoemission spectroscopy with polarized photon on FeSe/SrTiO_3_ and observe high-order replica bands with high intensity from various Fe 3*d* bands, suggesting a mixed mechanism.

## Introduction

Since its discovery in 2012^1^, monolayer (ML) FeSe grown on a SrTiO_3_ (STO) substrate has drawn much attention. In addition to a superconducting transition temperature (*T*_c_) over five times its bulk counterpart^1,2^, FeSe/STO stands out amongst all iron-based superconductors, with both the largest superconducting energy gap of ~15–20 meV and the highest gap opening temperature of ~60–70 K^2–4^. Previous angle-resolved photoemission spectroscopy (ARPES) work revealed that ML FeSe on STO is doped with ~0.12 electrons per Fe atom, transferred from the STO substrate^3,4^. Although a similar electron doping drives an enhanced superconductivity also in many other FeSe-based systems^5–7^, this mechanism alone was thought to be insufficient to explain the specifically large energy gap of FeSe/STO.

In pursuit of possible supplementary mechanisms by which the enhanced superconductivity in FeSe/STO is supported, interfacial electron-phonon coupling (EPC) has been of primary consideration^8–10^. This is largely due to the observation of replica bands in the ARPES data. In addition to the primary photoelectron spectra, secondary intensity peaks which replicate the dispersion of the former can be identified at higher binding energies by a shift of ~90–100 meV^8,11^. This is close to the energy of the STO’s longitudinal optical phonon or Fuch–Kliewer (FK) phonon mode^9,12^. Hence the replica bands were interpreted at first as the result of forward scattering of Fe 3*d* electrons by the STO phonons: a hallmark of strong EPC^13^. Recently the energy separation between the replica and principal peak was found to be slightly larger than the phonon energy, which was explained in the picture of EPC^14^. A connection was also drawn between the superconducting gap size and the replica peak amplitude, but this claim remains difficult to substantiate because of the complicated energy- and momentum-dependent photoelectron background signal.

Despite the attractive simplicity of the EPC argument, there are important concerns regarding the viability of this explanation for the replica features and their purported connection to high-*T*_c_ superconductivity^15^. One possible alternative considers extrinsic photoelectron energy loss processes^16^. In this scenario, the escaping photoelectrons interact with a time-dependent electric field from the STO substrate via a strongly coupled FK surface phonon at momentum *q* = (0, 0). This interpretation has support from high-resolution electron energy loss spectroscopy (EELS) performed on STO^12,17^ at comparable incident energies and angles to those used in the ARPES measurements. This would imply that there is no direct connection between EPC and the replica bands, although EPC may still be relevant to the material’s properties.

On the other hand, a bulk compound (TBA^+^)FeSe, where organic tetrabutyl ammonium molecules are intercalated in between FeSe layers, is reported to have a *T*_c_ of 43 K and a pseudogap up to 60 K^18^, comparable to FeSe/STO. The results have brought new debate on the role of the substrate in the superconductivity.

As FeSe/STO has demonstrated that interfacial engineering promises a viable route toward the enhancement of *T*_c_ in unconventional superconductors, an understanding of the physical mechanism involved is of central importance to the field. It is essential to ascertain whether the replica features observed in photoemission are indicative of EPC and its pivotal role in the physics of this material, or if this is merely an artefact of the experimental configuration detracting from the subject of interest.

To pursue this goal, further ARPES measurements are required. Challenges that have limited the progress come from not only the strict growth conditions that are necessary to ensure optimal superconductivity and clear replica bands^19–21^, but also the nature of the electronic states in FeSe/STO. On account of the strong correlations in this material^22^, the electron removal spectral function observed experimentally is broad, with extensive energy dependence, well beyond the sharp quasiparticle features familiar from bulk FeSe^23^. This is further complicated by the presence of a substantial energy and momentum-dependent background. Near the chemical potential, where several bands of distinct orbital characters disperse through the same regions of energy and momentum, the usual inelastic background signal is supplanted by the tails of spectral features associated with different electron bands. Considering the objective of resolving individual replica bands and their relative intensities, this represents a formidable practical challenge to the unambiguous characterization of the ARPES spectra in FeSe/STO.

In this work, to address this challenge, we carry out synchrotron-based ARPES measurements with high energy and momentum resolution, using linearly polarized photons. As the familiar dipole selection rules impose strict constraints on the spatial symmetry of the electronic eigenstates observed via photoemission^24,25^, the correct choice of polarization enables us to select specific bands of interest, suppressing contributions from nearby states entirely. Through application of this approach, we are able to observe replica bands at both the M and Γ points of the Brillouin zone (BZ), largely suppressing contributions from other nearby states. As the replica features are related to the excitation of a quantized bosonic mode, one should in principle observe higher-order replica features, with kinetic energies further reduced by multiples of the mode energy. Indeed, in addition to the first-order replica bands, we observe higher-order ones, and successfully extract the relative peak amplitudes. Without the complications of a pronounced background signal, we identify replica intensities in great excess of those reported previously, calling into questions about the physical origin of these features.

## Results

### ARPES on ML FeSe/STO with polarized photons

**Fig. 1:**
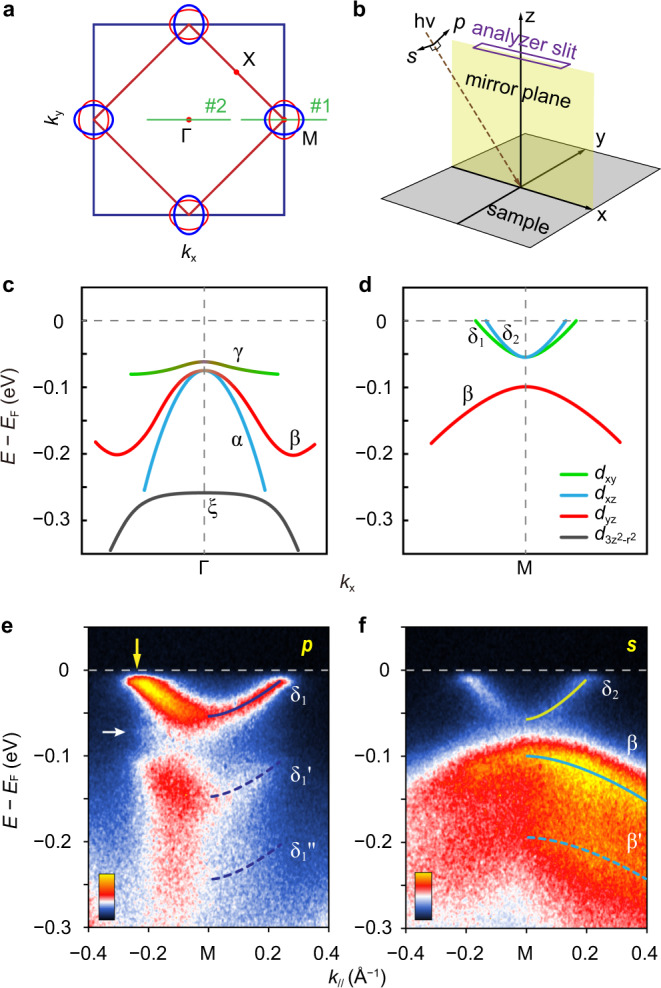
ARPES characterization with polarized photons. **a** The 2-Fe (red square) and 1-Fe (blue square) Brillouin zones (BZs), and the sketch of Fermi surfaces (blue and red ellipses) of monolayer FeSe/STO^26^. The green lines are the two cuts for the ARPES in Figs. 1–4. **b** Experimental geometry for linear polarization-dependent ARPES, where *p* (*s*) indicates the electric field of incident photon is parallel (perpendicular) to the emission plane defined by the analyzer slit. **c**, **d** Schematic diagram of band structure and orbital characters of monolayer FeSe/STO at Γ and M points, respectively, as determined by this ARPES study and in agreement with refs. ^26,27^. Here we use the same coordinate system as in **b** to define the *d* orbitals, i.e., *x* and *y* along the nearest Fe–Fe directions. Photoemission maps along cut #1, with 24 eV photons in *p* polarization (**e**) and *s* polarization (**f**). The solid and dashed curves indicate principal and replica bands with ~90 meV intervals, respectively. The white arrow indicates the other set of replica band at ~60 meV below the principal band. The yellow arrow indicates the Fermi momentum of the δ_1_ band, where a superconducting gap is opened.

As illustrated in Fig. 1a, in the 2-Fe unit cell imposed by the glide-plane of the FeSe crystal, the Fermi surface of FeSe/STO is characterized by two crossed elliptic pockets centered at the M point^26^. As the low-energy electronic structure is well represented by the bands near Γ and M, we focus our attention on the two cuts identified in Fig. 1a.

The ML FeSe films presented here are in the optimally doped and superconducting state (see “Methods” section, Supplementary Fig. 1, and ref. ^20^ for details of the sample growth). The doping level determined from the size of the electron pocket is ~0.11 electrons per Fe, while the superconducting gap is ~15.0 meV extracted from the energy distribution curve (EDC) at the Fermi momentum (Supplementary Fig. 2a), indicating high sample quality.

Our experimental geometry is sketched in Fig. 1b, where the emission plane coincides with the (110) mirror plane of FeSe lattice that is parallel to the nearest Fe–Fe direction, as confirmed by the low-energy electron diffraction (LEED) pattern (Supplementary Fig. 1c). The photoemission matrix element, dependent on the photon polarization and the initial and final electron wave functions, is important in determining the ARPES instensity^24^: for *p* (*s*) geometry where the photon polarization vector is parallel (perpendicular) to the mirror plane, only even (odd) initial wave functions will be detected. This provides an experimental means by which closely spaced bands of particular orbital characters or parities can be addressed or suppressed selectively in the photoemission signal. The selection rules lead to a pronounced suppression of spectral background, enabling improved characterization of the replica bands.

From previous ARPES measurements^26,27^, we expect four bands (α, β, γ and ξ) at Γ point (Fig. 1c) and three bands (δ_1_, δ_2_, and β) at M point (Fig. 1d) near Fermi level, consisting of different Fe 3*d* orbitals. In our *p*-polarized measurement, as shown in Fig. 1e, only one electron-type quasiparticle band δ_1_ is visible at M point. Two replica bands, δ_1_′ and δ_1_″, show up on top of a clean background, with similar dispersion to the principal band (see Fig. 1e). Due to the gap opening at the Fermi level, the band shows apparent back-bending behavior, which is directly inherited in the replica band (Supplementary Fig. 2b). δ_1_, δ_1_′, and δ_1_″ are separated by an energy of ~94 meV, corresponding to one of the FK phonon modes of STO^28^. A faint replica band is captured at ~60 meV below the principal band (marked by the white arrow in Fig. 1e), corresponding to the other FK phonon of STO^28^. δ_2_ and β bands, on the other hand, are probed with the *s* polarization in Fig. 1f. By considering the experimental geometry, the selection rules and the symmetry analysis of the *d* orbital wave functions of two inequivalent Fe sites with the mirror plane running through Se sites^29^, we identify the orbital characters to be *d*_xy_ for δ_1_, *d*_xz_ for δ_2_, and *d*_yz_ for β, as shown in Fig. 1d^22,27^. The synchronous polarization dependence of principal bands and their replicas rules out the suspicion that the replica bands are the quasiparticle bands of other orbitals^30^.

### High-order replica bands and their intensities

There are mainly two proposed mechanisms for the replica bands, intrinsic EPC^8^ versus extrinsic energy loss of photoemitted electrons^16^. In both scenarios, multiple discrete replica bands are expected. The energy of the *n*th replica lies at *E*_qp_ − *n* × *E*_phonon_, where *E*_qp_ is the energy of quasiparticle band and *E*_phonon_ the phonon energy, resulting from electrons exciting *n* phonons each. Τhe intensity of the *n*th replica band should follow the Poisson distribution with zero temperature approximation^31–33^:

1 $${I}_{n}/{I}_{0}={\eta }^{n}/n!,$$

where *I*_0_ is the principal band intensity, *I*_n_ is the *n*th replica intensity, and $\eta ={I}_{1}/{I}_{0}$ characterizes the interaction strength. At the sample temperature of 9 K, *k*_B_*T* = 0.8 meV is much smaller than the *E*_phonon_, which validates the Poisson distribution. Multiple replica bands have been reported experimentally for the two-dimensional electron gas of the anatase TiO_2_^34^ and SrTiO_3_^32,35^, but have not been evidenced in FeSe/STO so far.

**Fig. 2:**
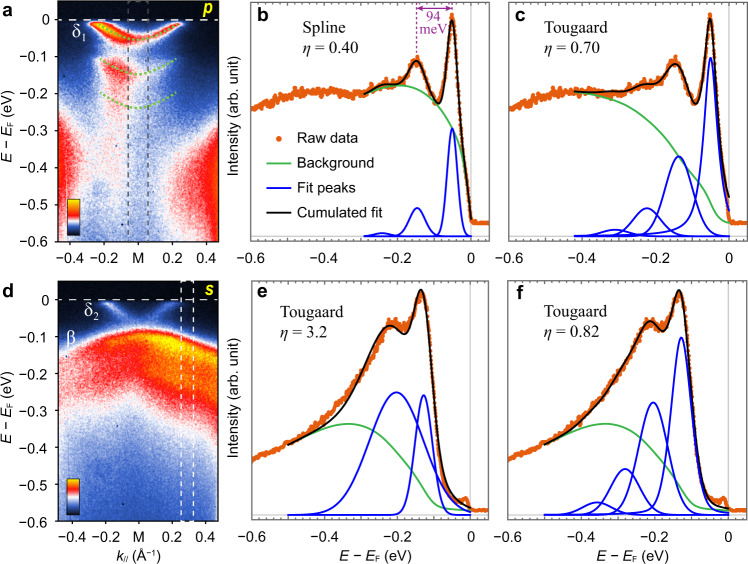
Replica bands in monolayer FeSe/STO at the M point. **a** Photoemission intensity along cut #1 in Fig. 1a, measured with 24 eV photons in *p* polarization. **b** Energy distribution curve (EDC) at M, integrated over the momentum range indicated by the dashed rectangle in **a**. The background is modeled using a cubic spline interpolation and the data are fit to three Gaussian peaks; *η* = *I*_1_/*I*_0_ is the ratio of the areas of the first replica and the principal peak. **c** Same as **b**, but with the EDC fit with a Tougaard-type background and multiple peaks. The peak areas follow the Poisson distribution ${I}_{n}/{I}_{0}={\eta }^{n}/n!$ (see “Methods” section for details). **d** Same as **a** but for *s* polarization. **e** EDC integrated over the momentum range indicated by the white dashed rectangle in **d** so that the δ_2_ band is excluded and only the β band contributes. The data are fit with a principal and a single replica peak which is unreasonably intense and broad. **f** Same as **e**, but with EDC now fit with a principal peak and three replica peaks, using the same method as in **c**.

Figure 2 shows ARPES results for δ_1_, δ_2_ and β bands at the M point and their replicas in a wider energy range. The δ_1_ band dominates the spectra at the M point with *p*-polarized light. In the integrated EDC (Fig. 2b), there is a principal peak and a pronounced replica peak with ~94 meV offset. The hump observed approximately at −0.25 eV is the hallmark of the second replica of the principal peak. Below −0.3 eV is a dome-shaped background whose intensity is strongly dependent on the photon energy and unrelated to the principal peak, as will be discussed later on. The tails and inelastic background from other bands constitute the majority of the signal in this regime, which requires subtraction from the total signal in order to estimate the peak intensity.

We first follow the background method that was used in refs. ^8,14^ with a spline interpolation curve that running though several anchor points at the local minima of the data (green line in Fig. 2b). The intensity ratio between the first replica peak and the principal peak *η* is fit to be 0.40, almost twice the largest value measured with unpolarized photons in ref. ^14^. With such a large *η* in Eq. (1), the second-order replica intensity should be *I*_2_ ≈ 0.08 *I*_0_, whereas the fitting result is *I*_2_ ≈ 0.04 *I*_0_. This suggests that using a spline interpolation for the background, which has no physical basis, is likely to be unsuitable for these data. In fact, there is not an apparent explanation for such a high, dome-shaped background in the energy range above −0.3 eV in the *p* polarization measurement, where the spectral weight from the β band has already been suppressed.

In pursuit of a more suitable and physically motivated choice, we adopt a Tougaard-type background with multiple-peak fitting (see “Methods” for details), as shown in Fig. 2c. The Tougaard background considers the inelastic scattering of the electrons, and is widely used in photoemission spectroscopy^36,37^. The spectral lineshape after background subtraction is nicely fit to a principal peak along with three replica peaks that follow the relation in Eq. (1) with *η* = 0.70. We cut off at the third replica band, as additional replica peaks will carry <3% of *I*_0_. The same methodology has been applied to all other multiple-replica fittings in this work.

In the *s* polarization measurement, both δ_2_ and β bands are manifested. Only the replicas of β band are visible while those of the δ_2_ band, which are much weaker, overlap with the β band. We consider an integrated EDC acquired at a momentum beyond the extent of the δ_2_ band to avoid its influence on the spectral fitting, as shown in Fig. 2d–f. The spectral shape is different from that of the δ_1_ band in Fig. 2b, clear of the dome-shaped background from other bands. In this case the spline background that attaches to the data points is not feasible, and we return to using the Tougaard background for peak fitting. Although the second replica peak cannot be directly recognized in the EDC, a single replica peak fails to fit the data and exhibits an unreasonably high intensity with *η* = 3.2 and an extremely large linewidth (Fig. 2e), whereas the higher-order replicas must be involved when *η* is large according to Eq. (1). Ultimately, we successfully fit the data using a Tougaard background plus one principal peak along with three replica peaks with *η* = 0.82 (Fig. 2f). The *η* for the β band is higher than that of the δ_2_ band, which holds true for different momentum windows being taken (Supplementary Fig. 3), implying a possible orbital dependence of the electron-phonon interactions.

**Fig. 3:**
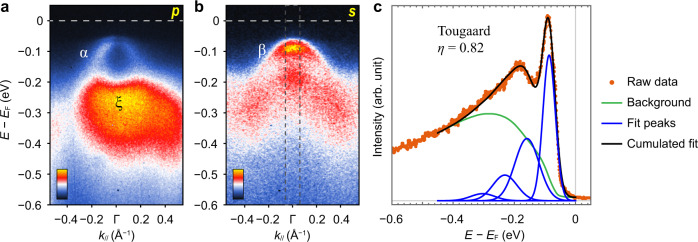
Replica bands in monolayer FeSe/STO at the Γ point. **a** Photoemission intensity along cut #2 as indicated in Fig. 1a, measured with 24 eV photons in *p* polarization. **b** Photoemission intensity along cut #2 in Fig. 1a, measured with 50 eV photons in *s* polarization. **c** EDC at Γ, integrated over the momentum range indicated by the dashed rectangle in **b**. The data are fit with the same method as in Fig. 2f.

Along cut #2 near the Γ point, several hole bands (α, β, and ξ) are observed (Fig. 3). In *p* polarization (Fig. 3a), the α band intensity is weaker and the strong ξ band lies below it, heavily superimposed over the replicas of the α band. By contrast, the α and ξ bands are eliminated with *s* polarization, while the β band and its replicas become most pronounced (Fig. 3b). The β band at Γ point can be fit similarly to our procedure at the M point and *η* is also 0.82 (Fig. 3c), adding confidence in this choice of fitting. Remarkably, the multiple replica features and the *η* value are similar to that observed on an annealed bare STO surface^32^, hinting at a common physical origin.

### Fitting of polarization-mixed data

**Fig. 4:**
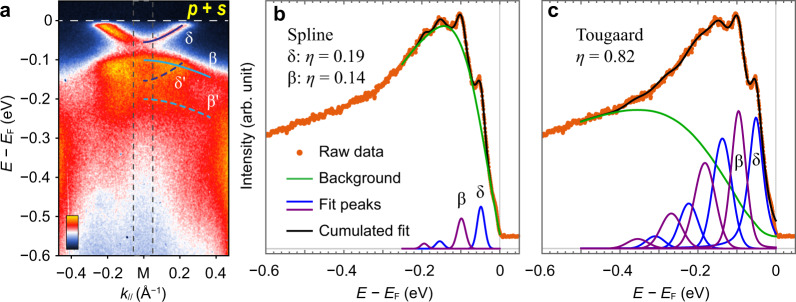
Fitting ARPES results with mixed polarization. **a** Sum of the APRES maps around M point taken in *p* and *s* polarizations (Fig. 2a, d). **b** EDC integrated over the momentum range indicated by the dashed rectangle in **a**. The background is modeled using a cubic spline interpolation. The data are fit to four Gaussian peaks, corresponding to δ and β bands and their replicas. **c** Same data as **b**, but fit with a Tougaard background and two sets of multiple peaks for the δ and β bands, respectively.

To further demonstrate the advantage of our polarization-dependent strategy, we contrast our fitting results against those derived from unpolarized data. To accommodate a direct comparison, we sum over the intensity maps from the *p* and *s* polarizations to mimic the data obtained with unpolarized light (Fig. 4a) and take the integrated EDC around the M point (Fig. 4b). The results become similar to those in ref. ^14^ obtained with unpolarized photons, indicating the role of the polarization-dependent background in the anomalously small values of *η* reported previously.

Due to the coexistence and overlapping of the δ and β bands in a narrow energy range, the peak features are obscured by the large total signal, ultimately resulting in an underestimation of the replica intensity *η* when the spline background is used for peak fitting. In line with ref. ^14^, the *η* extracted from polarization-mixed data is not larger than 0.2 (Fig. 4b), while *η* is ~ 0.4 and by a factor of two higher for the polarized data (Fig. 2b), both with spline background. On the other hand, although the Tougaard background plus two groups of peaks manage to fit the polarization-integrated data (Fig. 4c) with *η* = 0.82, which is similar to that in Fig. 2f, an undesirable increase in the number of parameters ensues due to the complicated spectral components. The contrast between Figs. 2 and 4 demonstrates the significance of photon-polarized ARPES for more reliable characterization on the replica bands of FeSe. We also note that the background method such as the spline that cannot yield consistent results between different measurements appears to be less suitable for quantitative and delicate analysis.

### Photon energy-/emission angle-dependent ARPES results

**Fig. 5:**
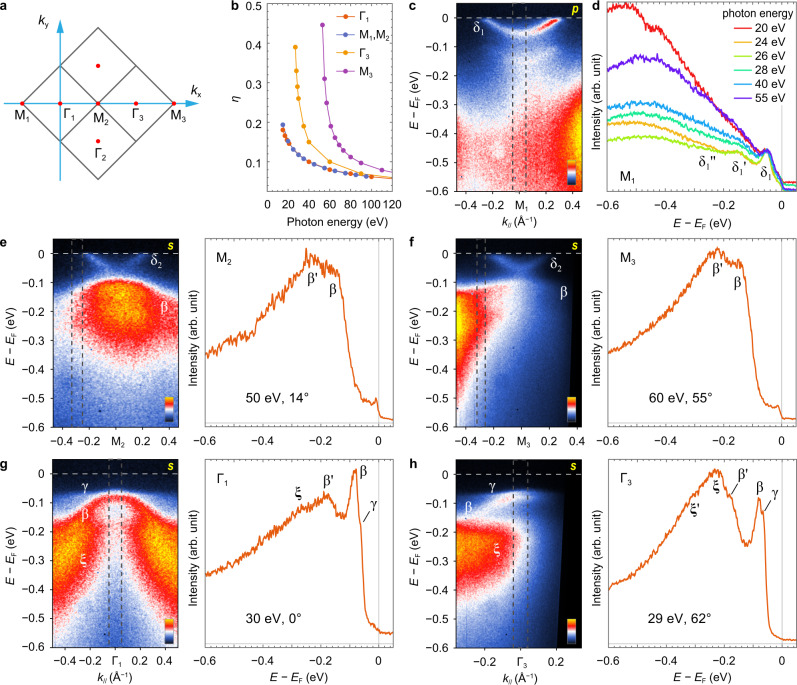
Photon energy/emission angle-dependent ARPES results showing substantial variation of the background shape. **a** Extended reciprocal space and high-symmetry points of FeSe. The squares are BZ boundaries. **b** Calculated photon energy-dependent replica intensity at selected high-symmetry points, according to electron energy loss scenario. **c** ARPES map around M_1_ point measured with *p*-polarized, 26-eV photons. **d** EDCs at M_1_ taken with various photon energies, integrated over the momentum range indicated by the dashed rectangle in **c**. The curves are normalized by the principal peak maximum, and the background shows large variation with photon energy. **e**–**h** ARPES results at M_2_, M_3_, Γ_1_, and Γ_3_, respectively, measured with *s-*polarized photons. For each of **e**–**h**, the left panel is the intensity map, and the right is the integrated EDC, labeled with the photon energy and the emission angle of the electrons with respect to the sample normal. All measurements were taken with the analyzer slit along *x* direction.

In the photoelectron energy loss process, the probability of generating an excitation is inversely proportional to the electron momentum perpendicular to the surface, *i.e*., $\eta \propto 1/{k}_{e}{{{\rm{cos }}}}(\theta )$, where *k*_*e*_ and *θ* are the wave vector and escaping angle of the emitted electron relative to the surface normal, respectively. *k*_*e*_ is dependent on incident photon energy *E*_photon_, and *θ* is dependent on both *k*_*e*_ and the measured in-plane momentum ${k}_{\parallel }$ in the reciprocal space. For the 94 meV FK phonon, the derived *η* for various *E*_photon_ and high-symmetry points of BZ (Fig. 5a) are shown in Fig. 5b. The replica intensity increases with decreasing *E*_photon_ or increasing *θ*, which is distinguished from intrinsic EPC scenario where no variation of *η* is expected.

In reality, however, although photon polarization has suppressed a great portion of background observed in previous experiments, we found that a complicated *E*_photon_-dependent (Fig. 5d) and *θ*-dependent (Fig. 5e–h) background persists, due to the neighboring *d* bands, the inelastic scattering of photoelectrons, or Debye–Waller effects on photoemission matrix elements^38^. In addition, the photoemission intensity drops off extremely fast in the high BZs approaching grazing angles (Fig. 5f, h). The unpredictable evolution of the background with measurement parameters makes it arduous to determine the intrinsic difference of replica band intensity for different *E*_photon_ or *θ*.

## Discussion

We have shown that the measurement conditions have great impacts on the photoemission spectra, and certain parameters are preferable to obtain high signal-background ratio for replica bands, as summarized in Table 1. The background model, the peak lineshape, and the fitting parameters still undoubtedly influence the extracted replica amplitude *η* reported here. On the other hand, our methodology with carefully chosen photon energy and polarization provides higher data quality. The *η* values exceed those reported previously (0.05–0.22) by a significant margin^14^, regardless of the background models. The high *η* value together with the high-order peaks indicates that the interaction from which the replica bands are derived must be strong. Furthermore, the replica intensity appears to have orbital dependence for *d*_yz_ band (β) and *d*_xy_ band (δ_1_). These findings require a reconsideration of the proposed interpretations of the replica bands and their relation to the enhanced superconductivity in FeSe/STO.

**Table 1:** Parameters for high-visibility replica bands in the first BZ.

Band	Position in BZ	Polarization	Photon energy	*η*
*d*_xy_ (δ_1_)	M point	*p*	24 eV	0.70
*d*_yz_ (β)	M point	*s*	24 eV	0.82
*d*_yz_ (β)	Γ point	*s*	50 eV	0.82

The high *η* value is unexpected from the existing EPC and photoelectron energy loss theories. For the interfacial EPC model, the replica band intensity *η* is approximately proportional to the dimensionless EPC constant *λ* for *λ* < 1^39,40^. According to Migdal–Eliashberg theory^40^, *λ* = 0.2 is enough to induce a *T*_c_ of 70 K, but the second-order replica bands should not be observable for *λ* below 0.3. For *η* = ~0.7–0.8, based on the quantum Monte Carlo simulation^39^, *λ* would be ~0.6–0.7, much higher than expected from the *T*_c_. In another theoretical study^15^, when Coulomb interaction amongst electrons in FeSe is taken into account, the phonon induced attractive potential is almost screened. This implies that the strong replica bands cannot be solely attributed to EPC. In the photoelectron energy loss process, the escaping electron can interact with STO phonons over a longer distance^16^, so the ~0.5-nm height of Fe atoms in ML FeSe relative to the STO surface^41,42^ is of little consequence here. This explains the comparable replica intensity measured from both ML FeSe/STO and bare STO. Nevertheless, the calculated *η* value for the measurements in the first Brillouin zoon is still smaller than 0.2 (Fig. 5b), distinct from our experimental results.

The different replica band intensity between *d*_yz_ and *d*_xy_ could be understood under EPC, when the electrons in the FeSe are coupled with the vertical dipole vibration of the oxygen atoms in STO^9^. On the other hand, photoelectrons undergo the energy loss process when traveling as plane waves in the vacuum subsequent to the electron removal event, so the orbital dependence is difficult to be settled in this framework. However, we cannot entirely exclude the possibility that the different *η* values between bands result from the contamination of the spectra by adjacent bands or systematic error of background modeling or fitting. It is also possible that in reality, EPC and photoelectron energy loss both exist, leading to stronger replica bands than expected by either mechanism alone, and the contribution from EPC could carry some orbital dependence while the photoelectron energy loss remains independent.

After submission of this manuscript, we were aware of a relevant work^43^ where replica bands of δ_1_ are studied and the two major observations are (1) the replica band intensity has no dependence on the photon energy and (2) the energy separation between the first replica and the principal band (98 meV) is larger than the phonon energy obtained from EELS (94 meV). We note that the background used there is spline and the 2nd replica is not included in the fitting, different from this work. Besides, we do not observe a blue-shift of replica band on the same band in our data. In fact, the FK phonon energy itself depends on doping level and surface treatment of the STO^12^ with a variation in the order of a few meVs. In EELS results, the replica peak energy of ML FeSe/STO is smaller than that of bare STO^12^. Moving forward, further meticulously designed and extensive experiments and careful data analysis are required to provide solid evidence for the origin of replica bands and their relation with superconductivity in ML FeSe/STO. We recommend photon-polarized measurements as a necessary starting point for future studies.

## Methods

### Sample preparation

0.05 wt% Nb:STO substrates (CrysTec GmbH) were etched in deionized water and 10% HCl, and annealed in O_2_ at 1120 °C for 4 h in a tube furnace. ML FeSe films were grown in a Veeco GenXplor MBE system by codepositing Fe and Se on the substrates held at 420 °C^20^. The flux ratio Φ_Fe_:Φ_Se_ ≈ 1:5. The base pressure of the chamber was ~1 × 10^−10^ Torr. Reflection high-energy electron diffraction was used to monitor the sample quality (Supplementary Fig. 1a, b). The samples were annealed at 480 °C for 3 h and then cooled down to room temperature and capped with 15-nm-thick Te and 5-nm-thick Se layers before being exposed to air. Before the ARPES measurements, in order to remove the capping layers and achieve the optimal superconducting state, the samples were gradually annealed to 450 °C and kept for ~5 h in the preparation chamber at pressure lower than 1 × 10^−9^ Torr. LEED patterns and X-ray photoelectron spectroscopy data were collected to verify the film quality.

### ARPES

ARPES measurements were carried out on the Quantum Materials Spectroscopy Centre beamline at the Canadian Light Source, with vertically and horizontally polarized photons with energy ranging from 20 to 60 eV (see figure captions). Samples were measured at pressure lower than 5 × 10^−11^ Torr and a temperature of 9 K. The combined beamline-analyzer (Scienta R4000) resolutions in angle and energy are better than 0.1° and 9 meV, respectively.

### EDC fitting

The superconducting gap is determined by fitting the symmetrized EDC to a spectral function with the simplified BCS self-energy^44^ ${{\Sigma}} \left({{{\bf{k}}}},\omega \right)=-{{{\rm{i}}}}{\Gamma }_{1}+{{{\rm{{\Delta }}}}}^{2}/\left[\omega +\varepsilon \left({{{\bf{k}}}}\right)+{{{\rm{i}}}}{\Gamma }_{0}\right]$, where *ω* is the energy relative to Fermi energy, Δ is the gap size, *Γ*_0_ is the inverse pair lifetime, *Γ*_1_ represents the single-particle scattering rate, and $\varepsilon \left({{{\bf{k}}}}\right)$ is band dispersion [$\varepsilon \left({{{{\bf{k}}}}}_{{{{\bf{F}}}}}\right)=0$]. For each of Figs. 2c, e, f, 3c, and 4c, the Tougaard-type background $T\left(E\right)$ is computed by the convolution of the EDC data $S(E^{\prime} )$ and a Gaussian energy loss cross-section function: $T\left(E\right)=A{\int }_{E}^{\infty }{{{\rm{exp }}}}\left[-\frac{{\left({E}^{{\prime} }-E\right)}^{2}}{2{\sigma }^{2}}\right]S(E^{\prime} ){{{\rm{d}}}}{E}^{{\prime} }$, where *A* is the normalization factor and *σ* is the parameter that determines the shape of the curve. The data are fit to multiple peaks whose areas are constrained to follow the Poisson distribution ${I}_{n}/{I}_{0}={\eta }^{n}/n!$, where *I*_0_ is the area of the principal peak and *I*_n_ is the area of the *n*th replica. The peak positions are constrained as ${E}_{n}={E}_{0}-n\cdot \triangle E$. The principal peak is fit with a Gaussian–Lorentz summed function. The replica peaks are fit with Gaussian functions. The full width at half maximum is constrained to be the same for the replica peaks of each band. The standard error of the estimate for *η* is smaller than 0.02 in all fittings.

## Supplementary information

Supplementary Information

## Data Availability

All data supporting the key findings of this study are available within the article and its Supplementary Information. All raw data generated during the current study are available from the corresponding authors on reasonable request.
