# Supplementary material for: High-order replica bands in monolayer FeSe/SrTiO3 revealed by polarization-dependent photoemission spectroscopy
Source: Nat Commun. 2021 Jul 28;12:4573. doi: 10.1038/s41467-021-24783-5 (PMC8319137; doi:10.1038/s41467-021-24783-5)
Supplement: Supplementary file 1 — Supplementary Information [file 41467_2021_24783_MOESM1_ESM.pdf]

Supplementary Information

High-order replica bands in monolayer FeSe/$SrTiO_3$ revealed by polarization-dependent photoemission spectroscopy

Chong Liu[1,2], Ryan P. Day[1,2], Fengmiao Li[1,2*], Ryan L. Roemer[1,2], Sergey Zhdanovich[1,2], Sergey Gorovikov[3], Tor M. Pedersen[3], Juan Jiang[4], Sangjae Lee[5], Michael Schneider[1,2], Doug Wong[1,2], Pinder Dosanjh[1,2], Frederick J. Walker[4,5], Charles H. Ahn[4], Giorgio Levy[1,2], Andrea Damascelli[1,2], George A. Sawatzky[1,2] & Ke Zou[1,2*]

[1] Quantum Matter Institute, University of British Columbia, Vancouver, British Columbia V6T 1Z4, Canada.

[2] Department of Physics & Astronomy, University of British Columbia, Vancouver, British Columbia V6T 1Z1, Canada.

[3] Canadian Light Source, Saskatoon, Saskatchewan S7N 2V3, Canada.

[4] Department of Applied Physics and Center for Research on Interface Structures and Phenomena, Yale University, New Haven, Connecticut 06511, USA

[5] Department of Physics, Yale University, New Haven, Connecticut 06520, USA

* email: fengmiao.li@qmi.ubc.ca; kzou@phas.ubc.ca.

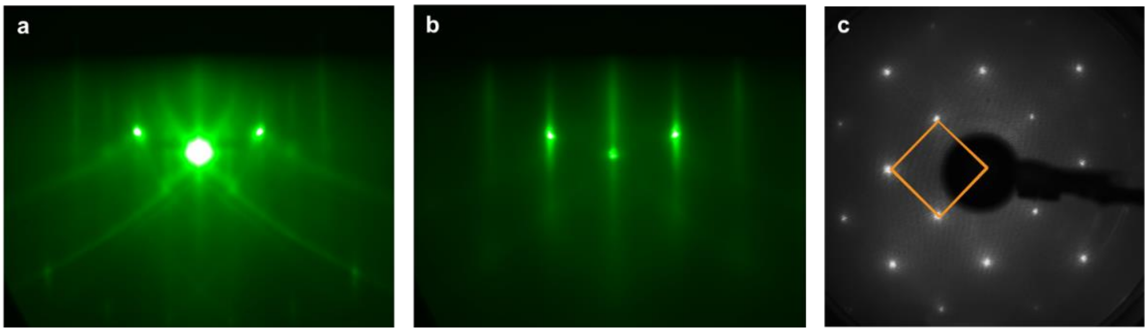

Supplementary Fig. 1 Electron diffraction of the substrate and monolayer FeSe film. a Reflection high-energy electron diffraction (RHEED) image of treated $SrTiO_3$ substrate with the incident beam along the [100] direction. The surface exhibits a c(4×2) reconstruction. Beam energy is 10 keV and emission current is 0.2 μA. **b** RHEED image of a monolayer FeSe film with the incident beam along the [100] direction. **c** Low-energy electron diffraction (LEED) image of a monolayer FeSe sample after decapping and annealing before ARPES measurement. Beam energy is 150 eV and emission current is 93 μA. The orange square indicates the 2-Fe unit cell in reciprocal space.

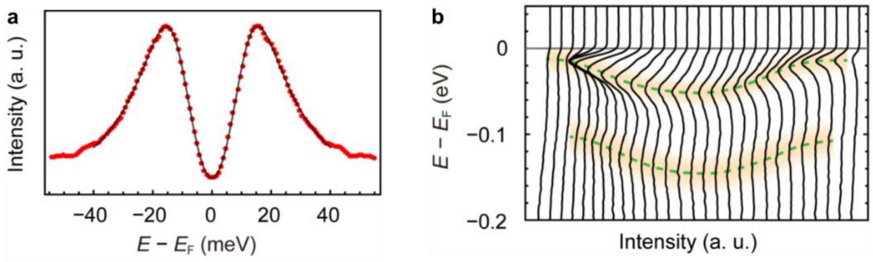

Supplementary Fig. 2 Superconducting gap and band back-bending. a Symmetrized energy distribution curve (EDC) (red dots) at the Fermi momentum of the δ_1 band marked by the yellow arrow in Fig. 2e. The black curve is the result of a fit based on the BCS self-energy with gap size of 15.0 meV. **b** EDCs of the data in Fig. 1e. The dashed lines and yellow shading trace the peak positions and highlight the band dispersions, which show back-bending near Fermi level.

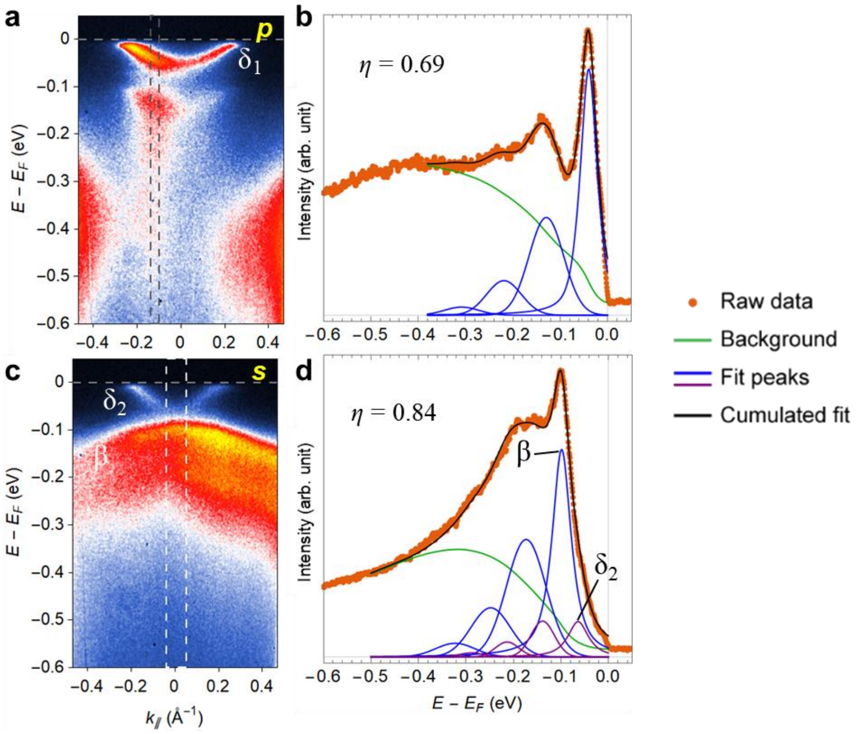

Supplementary Fig. 3 Fitting the same ARPES maps as Fig. 2 with different integration windows. **a** Photoemission intensity along cut #1 in Fig. 1a, measured with 24 eV photons in *p* polarization. **b** Energy distribution curve (EDC) at M, integrated over the momentum range indicated by the black dashed rectangle in panel a. The EDC is fit with a Tougaard-type background and multiple peaks. **c** Same as panel **a** but for *s* polarization. **d** EDC integrated over the momentum range indicated by the white dashed rectangle in panel c. Since both δ_2 and β bands are involved in this momentum window, two sets of multiple peaks are included on top of the Tougaard background in the fitting.
